# Endurance rivalry and female choice jointly influence male mating success in the emerald treefrog (*Zhangixalus prasinatus*), a lek-chorusing anuran

**DOI:** 10.1186/s40850-022-00117-w

**Published:** 2022-04-01

**Authors:** Yuan-Cheng Cheng, Yi-Huey Chen, Chunwen Chang, Ming-Feng Chuang, Yuying Hsu

**Affiliations:** 1 Department of Life Science, National Taiwan Normal University, No. 88, Sec. 4, Tingchou Rd., Wenshan Dist, Taipei, 11677 Taiwan; 2 Biodiversity Program, Taiwan International Graduate Program, Academia Sinica, and National Taiwan Normal University, No. 128, Sec. 2, Academia Rd., Nankang Dist, Taipei, 11529 Taiwan; 3 Department of Life Science, Chinese Culture University, No. 55, Hwa-Kwang Rd, Taipei City, 11114 Taiwan; 4 Division of Technical Service, Taiwan Forestry Research Institute, No. 53, Nanhai Road, Taipei City, 10066 Taiwan; 5 Department of Life Sciences and Research Center for Global Change Biology, National Chung Hsing University, 145 Xingda Rd., South Dist, Taichung City, 40227 Taiwan

**Keywords:** Endurance rivalry, Female choice, Lek attendance, Mating success, Sexual selection, Rhacophorid treefrog

## Abstract

**Background:**

Endurance rivalry and female choice are two important mechanisms of sexual selection in lek-breeding species. Endurance rivalry is when males compete for opportunities to mate by spending more time in leks than others (interaction-independent male-male competition). Because high-quality males can afford to have high lek attendance, females have a higher chance of mating with good-quality males even when they mate randomly. The good gene hypothesis proposes that females can pass good genes on to their offspring by choosing males that display elaborate morphological and/or behavioral traits that reflect the males’ genetic quality. The relative importance of lek attendance and female choice to males’ mating success in anurans is rarely evaluated. In this study, we investigated how these two mechanisms might jointly shape males’ morphological traits in the lek-chorusing emerald treefrog *Zhangixalus prasinatus*.

**Results:**

Our results show that (1) male lek attendance is positively correlated with body size and condition, and males with higher lek attendance have higher mating success, (2) the dominant frequency of males’ advertisement calls are negatively correlated with body size and males producing lower frequency calls have higher mating success, (3) male body size, but not body condition, has a non-significant positive relationship with mating success and (4) females show preference for calls with lower dominant frequencies in two-choice playback.

**Conclusions:**

Overall, both endurance rivalry and female choice play an important role in the mating success of male emerald treefrogs in the field and both are influenced by male body size/condition. By mating with males that have higher lek attendance and produce lower frequency calls, selection may indirectly favor larger males.

**Supplementary Information:**

The online version contains supplementary material available at 10.1186/s40850-022-00117-w.

## Background

Sexual selection can operate through male-male competition (intra-sexual selection) and female choice (inter-sexual selection) to affect male reproductive success [1–3] and shape male traits [1, 4]. Male-male competition may facilitate or limit/hinder the expression of female choice [5–7]. For instance, in the three-spined stickleback (*Gasterosteus aculeatus*), male red breeding coloration functions both as a threat signal in male-male competition and as a trait for female choice: the difference in the intensity of the signal between the males increases after competition, which facilitates female choice and results in a preference for dominant males [8]. In the tiger salamander (*Ambystoma tigrinum tigrinum*), however, the two mechanisms favor different male traits: although females prefer males with long tails, males with larger body length have an advantage in male-male competition and are more likely to interrupt courting pairs [9]. Because these two mechanisms often do not act independently, a more comprehensive view of the evolution of sexually selected traits can be obtained by studying them simultaneously [5].

In lek-breeding systems, males aggregate and display to females that then choose among them [10]. Large variation in male mating success is frequently observed in lek-breeding species [11, 12]. Endurance rivalry and female choice are two important mechanisms of sexual selection in lek-breeding systems that contribute to the variation in male mating success [1, 13–15].

Endurance rivalry, interaction-independent male-male competition [16], is when males compete for opportunities to mate by spending more time attending leks [13, 16, 17]. Endurance rivalry is usually measured as the number of days per season males display in leks (i.e., lek attendance). Positive correlations between lek attendance and male mating success have been reported for several amphibians [18, 19] and birds [20–22]. Attending leks is time-consuming and energetically costly, which decreases the time and energy that can be allocated to other activities such as foraging [23]. Males’ ability to attend leks therefore can reflect their physical condition or stamina and, to some extent, their genetic quality [19]. Because high-quality males can afford to participate in leks more frequently and/or for longer periods, females have a higher chance of mating with good-quality males even when they mate randomly [19].

Females’ preferences for males with exaggerated morphological traits (e.g., longer tails in Jackson's widowbirds *Euplectes jacksoni*, [24, 25]) or extravagant displays (e.g., longer call duration in gray treefrogs *Hyla versicolor*, [26, 27]) have been found in a wide range of taxa [10]. According to the good gene hypothesis, females may benefit from mating with males with exaggerated morphological traits or extravagant displays [28]. In most lek-breeding species, males provide females with no resources other than sperm, and females acquire only genetic (indirect) benefits from mate choice [28, 29]. Given that quality is heritable, females that mate with males with high genetic quality will produce offspring with high genetic quality [28, 30]. Because ornaments and advertisement displays are costly to produce, they could serve as honest signals for good quality in males [31–33]. Consequently, females that choose to mate with males with these traits will pass ‘good genes’ on to their offspring [33–35].

In lek-chorusing frogs, males aggregate near to water ponds and produce advertisement calls (usually forming a chorus) to attract females [36]. Several studies have shown that male frogs that spend more nights in the leks are more likely to acquire mates [17]. Moreover, females often choose males based on the characteristics of their calls [26, 37–39] and these call properties reflect the morphological traits (e.g., body size, body condition) of the calling males [40–42]. Call rate and duration are considered honest signals of male quality in some species because producing more frequent calls with longer duration is energetically demanding [43, 44]. The dominant frequency of calls is an honest signal of male quality because it is usually negatively associated with body size [40, 42] and larger males tend to grow faster and/or survive longer [45, 46]. Because of their prolonged breeding season [47] and the relative ease of handling and carrying out field and laboratory experiments, lek-chorusing anurans have been used in many studies to evaluate the importance of endurance rivalry or female choice to male mating success [17, 37, 38] and the evolution of male morphological traits (e.g., [48, 49]). In *Physalaemus pustulosus* [48] and *Hyla chrysoscelis* [49], for instance, female preference for low frequency calls results in selection for larger males. Despite these findings, the relative importance of endurance rivalry and female choice in influencing male mating success and hence the evolution of male morphological traits remains unclear. A few studies have examined the two mechanisms simultaneously. In the European treefrog (*Hyla arborea*), chorus attendance influenced males’ mating success [19], but females did not show a directional preference for any of the properties of males’ advertisement calls [50], suggesting that endurance rivalry is an important mechanism of sexual selection in the species, but that female choice is not. In the Italian treefrog (*Hyla intermedia*) [18], both male chorus attendance and call rate influenced male mating success (although chorus attendance explained more variation in mating success than call rate). Furthermore, body condition was significantly associated with lek attendance and predicted mating success. The study concluded that selection on chorus attendance and calling quality may indirectly result in selection for males with higher body condition [18]. Overall, the relative importance of endurance rivalry and female choice in influencing mating success and hence the evolution of male morphological traits (body size and body condition) in lek-chorusing frogs appears to differ between species.

In anurans, there could be trade-offs between attending leks and producing attractive calls. Attending leks for extended periods increases a male’s chance of encountering a female but also increases energy consumption [36, 51, 52]. In lek-chorusing frogs, a male’s ability to attract a female often depends on the rate and/or duration of its calls [53], and calls of higher rates and longer duration are usually more energetically demanding [43, 44]. A male may therefore have to apportion available energy to these two behavioral tactics proportionally during breeding seasons to maximize its mating success. Examining the role of lek attendance and female choice in determining male mating success provides insights into how sexual selection operates in the population and whether endurance rivalry and female choice result in selection for similar morphological traits.

**Fig. 1:**
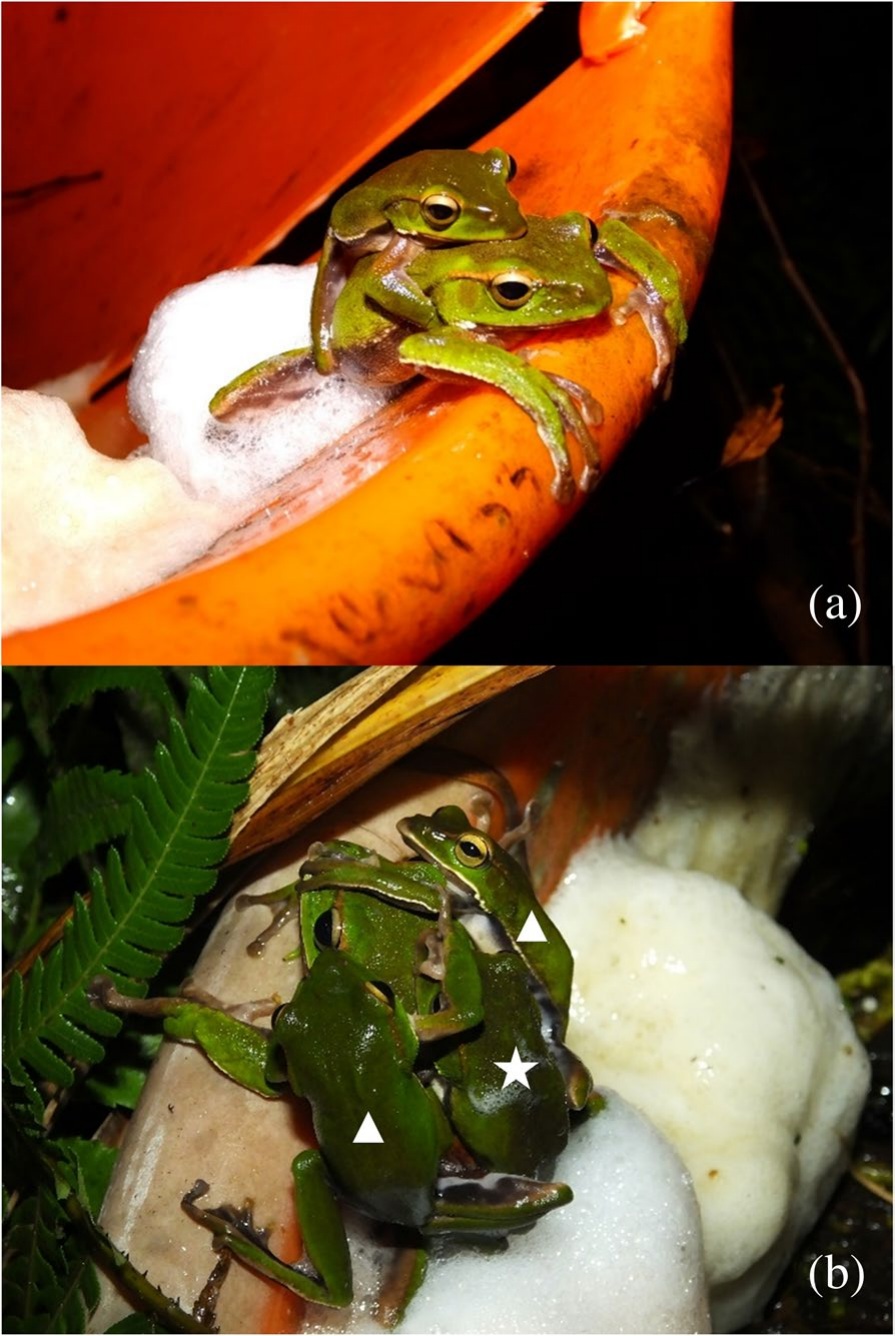
Amplexus of the emerald treefrog *Z. prasinatus*. **a** One female mates with one (primary) male (monandrous mating), and **b** one female mates with one primary male (★) and two peripheral males (▲) (polyandrous mating)

In this study, we examined the importance of endurance rivalry and female choice in influencing mating success and hence the evolution of male morphological traits (body size and body condition) in a population of emerald treefrogs (*Zhangixalus prasinatus*). The emerald treefrog is a lek-chorusing treefrog endemic to Taiwan [54–56]. Males of *Z. prasinatus* gather on plants surrounding water bodies, produce advertisement calls and form choruses at night to attract females [54]. All males that appear near water bodies produce advertisement calls, and no silent satellite behavior has ever been observed in this species [54]. A mating event starts with a female entering the chorus arena and approaching a male (the primary male). The (primary) male then grasps the female’s axillary region with his forelimbs (amplexus) and the female carries the male to an oviposition site. Oviposition usually occurs on the plants next to natural ponds or above the waterline on the inner wall of artificial containers. The female releases mucus from its cloaca, uses its hind limbs to whip and blend the mucus into a foam nest and then releases eggs into the foam nest (Fig. 1a). Polyandrous mating has been found in this species. During the oviposition process, unpaired (peripheral) males sometimes gather around the mating pair and jump on the amplectant pair which can result in simultaneous polyandry (Fig. 1b). The paternity shares of the primary and peripheral males in *Z. prasinatus* are currently unknown. However, a study of a closely related species (Omei treefrog, *Zhangixalus omeimontis*, renamed from *Rhacophorus omeimontis to Z. omeimontis* in 2019 [57]), showed that the primary male obtains the majority of the paternity share (> 70%) in polyandrous mating [58]. Moreover, for the multimale amplexus in the Australian frog *Crinia georgiana*, (1) males that spent more time amplexed had greater fertilization success, and (2) males that were in the dorsal position had a fertilization advantage over males in alternative positions, fertilizing on average 50% of a female’s clutch [59]. The primary males in our study were the ones to first form amplexus with the females and also the ones to assume the dorsal position. We therefore expect, in polyandrous mating in our study species, the primary males to have a much higher paternity share than the peripheral males (primary males > peripheral males). Physical fights (i.e., direct male-male competition) between males have never been observed (personal observations), which highlights the potential importance of endurance rivalry and female choice in determining male mating success. In this study, we conducted a field survey to (1) examine the effect of male body size and body condition on mating success, (2) examine the effect of male lek attendance on mating success and the relationships between lek attendance and morphological traits. We also used data from field observations to (3) examine the effect of male call properties on mating success and the relationships between call properties and male morphological traits. We further examined the relationship between male lek attendance and call properties to (4) detect potential trade-offs between male ability to attend leks and to produce attractive calls. Furthermore, we conducted controlled experiments to (5) confirm female preference for the call properties found to influence male mating success in the field.

## Results

We caught and marked a total of 235 males and observed 66 mating events. Out of the 66 mating events, 45 (68%) were monandrous and 21 (32%) were polyandrous. (10 of them involved 2 males, 7 involved 3 males, 3 involved 4 males and 1 involved 8 males.) Out of the 235 marked males, 153 were never observed to be involved in any mating event. For the 82 males that were observed to play a part in mating, 59 were observed to be the primary male at least once and 23 were only observed to be peripheral males. For the 59 primary males, 52 and 7 were observed to be the primary male in one and two mating events, respectively. Moreover, 2 and 11 out of these 59 primary males were also observed to participate once and twice, respectively, in other mating events as peripheral males.

### Males’ SVL had a non-significant positive relationship with mating success

Males’ mating success was categorized into three groups, having never been observed to play parts in mating events (unmated males, *N* = 153), having only been observed to participate in mating events as peripheral males (peripheral males, *N* = 23), or having been observed to be the primary male or the only male in at least one mating event (primary males, *N* = 59). The unmated males should have no paternity share. The primary males should have a much higher paternity share than the peripheral males because (1) the majority (68%) of the mating events were monandrous and (2) the primary males of polyandrous mating should have a higher paternity share than the peripheral males as explained in the Background. Because of the expected ordinal relationship in the paternity share of these three groups of males (mating success: primary males > peripheral males > unmated males), ordinal logistic regression models were used throughout this study to evaluate the importance of various factors to mating success.

We first examined the importance of SVL and body condition (the residuals of a male’s body mass regressed on its SVL) to male mating success. Ordinal logistic models showed that SVL had a non-significant positive association (L-R *χ*^2^_1_ = 2.98, *P* = 0.085, Table 1a), while body condition (L-R χ^2^_1_ = 0.90, *P* = 0.342, Table 1b) had no relationship, with male mating success.

**Table 1:** The importance of males’ SVL, body conditon and lek attendance to their mating success

	Variables	***b***	95% CI	df	L-R ***χ***^***2***^	***P***
(a)	SVL	0.09	(− 0.01, 0.19)	1	2.98	0.085
(b)	Body condition	0.14	(−0.15, 0.42)	1	0.90	0.342
(c)	Attendance	12.21	(8.17, 16.54)	1	37.78	**< 0.001**

Simple ordinal logistic regression models evaluating, separately, the importance of males’ (a) SVL, (b) body condition and (c) lek attendance to their mating success (i.e., being primary, peripheral or unmated males). *N* = 235 for all models. *P* values ≤0.05 are marked in bold

### Males with higher lek attendance had higher mating success

**Fig. 2:**
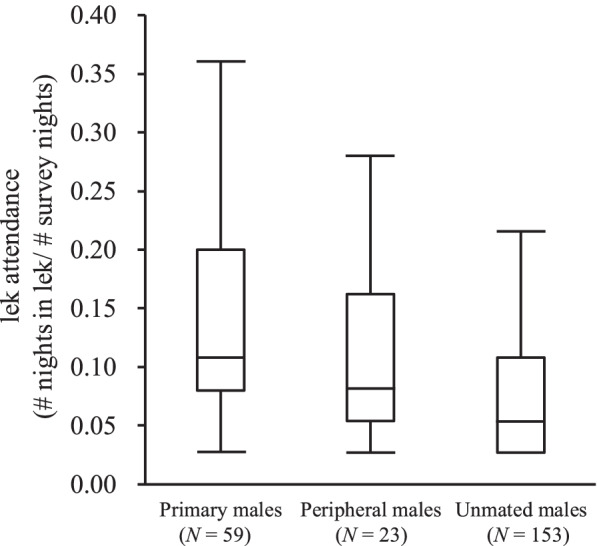
The lek attendance (box plots) of the primary, peripheral and unmated males. The line inside the box is the median, the bottom and top of the box refer to the 1st and 3rd quartiles

Male lek attendance was calculated as the number of nights the male was observed calling at a lek divided by the number of survey nights. An ordinal logistic regression model showed that male lek attendance significantly and positively predicted mating success (L-R *χ*^2^_1_ = 37.78, *P* <  0.001, Table 1c; Fig. 2); males that had higher lek attendance tended to have higher mating success than males that attended a lek on fewer nights.

Male lek attendance positively correlated with both SVL (*r*_*s*_ = 0.32, *N* = 235, *P* <  0.001) and body condition (*r*_*s*_ = 0.33, *N* = 235, *P* < 0.001). Males that were larger and in better condition spent more nights calling than males that were smaller and in poorer condition.

### Males that produced advertisement calls with lower dominant frequencies had higher mating success

**Fig. 3:**
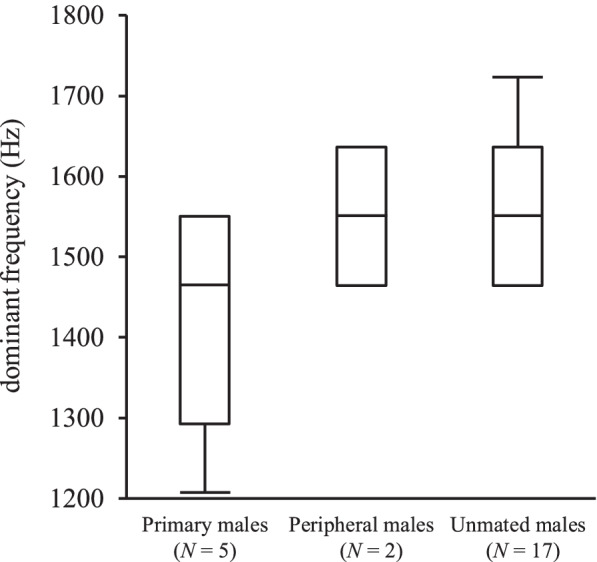
The call dominant frequency (box plots) of the primary, peripheral and unmated males. The line inside the box is the median, the bottom and top of the box refer to the 1st and 3rd quartiles

The calls of 24 males were successfully recorded; 5 were observed to be the primary male at least once, 2 were only observed to be peripheral males and 17 were never observed to mate. Male *Z. prasinatus* can produce three types of notes (types A, B and C). We analyzed the call properties of only type A notes, because all males produced type A but not type B or C notes. We quantified ten call properties, (1) note duration_adj_, (2) note rate, (3) note interval, (4) rise time_adj_, (5) fall time, (6) pulse number, (7) pulse rate_adj_, (8) dominant frequency, (9) Frequency modulation (FM)-rise and (10) FM-fall (explained in detail in “*Male calls*” and “Statistical analysis” in Methods). Ordinal logistic regression models showed that, out of the 10 call properties examined, only the dominant frequency of type A notes (L-R *χ*^2^_1_ = 5.29, *P* = 0.021, Table 2; Fig. 3) had a significant relationship with mating success; males that produced calls with lower dominant frequencies tended to have higher mating success.

**Table 2:** The importance of males’ call properties to their mating success

	Variables	***b***	95% CI	df	L-R ***χ***^***2***^	***P***
(a)	Duration_adj_	−4.25	(−41.98, 29.49)	1	0.06	0.807
(b)	Rate	1.13	(−1.61, 3.85)	1	0.73	0.394
(c)	Interval	−0.38	(−1.75, 0.86)	1	0.36	0.551
(d)	Rise time_adj_	14.67	(−26.17, 59.44)	1	0.49	0.482
(e)	Fall time	−48.40	(−142.47, 30.45)	1	1.40	0.236
(f)	Pulse number	−0.29	(−1.43, 0.67)	1	0.34	0.561
(g)	Pulse rate_adj_	−0.06	(−0.45, 0.32)	1	0.09	0.764
(h)	Dominant frequency	−0.01	(−0.02, − 0.00)	1	5.29	**0.021**
(i)	FM-rise	−0.01	(−0.02, 0.00)	1	2.05	0.152
(j)	FM-fall	0.00	(−0.00, 0.01)	1	1.81	0.178

Simple ordinal logistic regression models (a-j) evaluating the importance of each of the males’ call (type A notes) properties to their mating success (i.e., being primary, peripheral or unmated males). *N* = 24 for all models. *P* values ≤0.05 are marked in bold

Some, but not all, of the call properties significantly correlated with males’ SVL and/or body condition (Table 3). The dominant frequency (*r* = − 0.56, *P* = 0.004) and FM-rise (*r* = − 0.44, *P* = 0.032) of type A notes correlated negatively with males’ SVL. The duration_adj_ (*r* = − 0.45, *P* = 0.026), rise time_adj_ (*r* = − 0.43, *P* = 0.037) and pulse number (*r* = − 0.49, *P* = 0.015) of type A notes correlated negatively with males’ body condition.

**Table 3:** Pairwise correlations between males’ call properties and their SVL and body condition

	SVL		Body condition	
Variable	***r***	***P***	***r***	***P***
Duration_adj_	−0.30	0.152	−0.45	**0.026**
Rate	0.11	0.599	0.31	0.144
Interval	0.05	0.835	−0.13	0.549
Rise time_adj_	−0.26	0.216	−0.43	**0.037**
Fall time	−0.10	0.647	−0.28	0.189
Pulse number	−0.11	0.606	−0.49	**0.015**
Pulse rate_adj_	0.11	0.601	0.24	0.254
Dominant frequency	−0.56	**0.004**	−0.06	0.795
FM-rise	−0.44	**0.032**	−0.10	0.644
FM-fall	−0.11	0.597	0.18	0.403

*P* values ≤0.05 are marked in bold (*N* = 24)

**Fig. 4:**
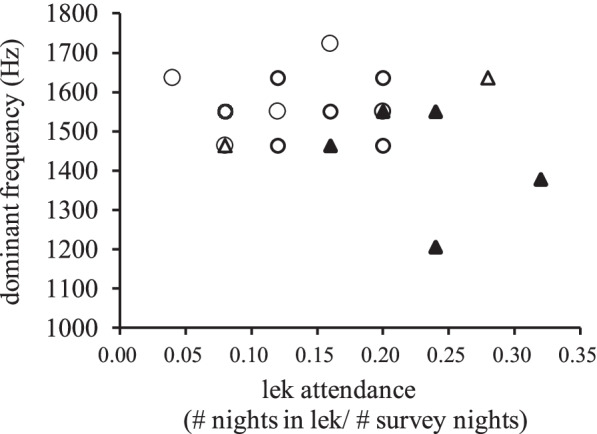
Males’ mating success in relation to both their call dominant frequency and lek attendance. ▲: primary males, △: peripheral males, ○: unmated males (lighter circle represents 1 male, bold circle represents 2 males)

To examine the importance of the dominant frequency of male calls to males’ mating success when lek attendance had already been considered, we constructed an ordinal logistic regression model including both factors. The results showed that both dominant frequency (L-R χ^2^_1_ = 4.83, *P* = 0.028) and lek attendance (L-R χ^2^_1_ = 6.78, *P* = 0.009) (Table 4) contributed to male mating success; males that produced lower frequency calls and had higher lek attendance tended to have higher mating success (Fig. 4). Although both lek attendance and dominant frequency were important to male mating success, these two traits did not significantly correlate with each other (*r*_*s*_ = − 0.11, *P* = 0.616; Table 5). Male lek attendance, however, correlated positively with note rate (*r*_*s*_ = 0.49, *P* = 0.015) and negatively with note interval (*r*_*s*_ = − 0.50, *P* = 0.013) (Table 5).

**Table 4:** The joint influences of call dominant fequency and lek attendance on males’ mating success

Variables	***b***	95% CI	df	L-R ***χ***^***2***^	***P***
Dominant frequency	−0.01	(−0.04, − 0.00)	1	4.83	**0.028**
Lek attendance	22.40	﻿(4.86, 48.87)	1	6.78	**0.009**

Multiple ordinal logistic regression evaluating the joint influences of males’ dominant frequency of type A notes and lek attendance on their mating success (i.e., being primary, peripheral or unmated males) (*N* = 24). *P* values ≤0.05 are marked in bold

**Table 5:** Pairwise correlations between males’ call (type A note) properties and their lek attendance

Variable	***r***_***﻿s***_	***P***
Duration_adj_	−0.34	0.105
Rate	0.49	**0.015**
Interval	−0.50	**0.013**
Rise time_adj_	−0.10	0.630
Fall time	−0.18	0.390
Pulse number	−0.20	0.341
Pulse rate_adj_	0.22	0.295
Dominant frequency	−0.11	0.616
FM-rise	−0.20	0.360
FM-fall	0.18	0.400

Spearman correlations were calculated because the distribution of lek attendance was very skewed (*N* = 24). *P* values ≤0.05 are marked in bold

### Females prefer calls with lower dominant frequency

**Fig. 5:**
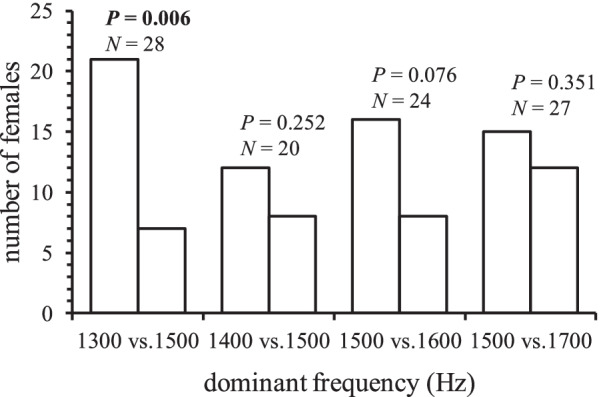
Females’ responses to call stimuli in the four two-choice playback tasks. *P* values ≤0.0125 are marked in bold (Bonferroni correction, 0.05/4 = 0.0125; one-tail binomial test)

Because call dominant frequency was significantly related to male mating success, we conducted four two-choice playback experiments where we gave females a choice between calls with a dominant frequency equal to the mean of the population (1500 Hz) and calls with a higher or lower dominant frequency: 1500 Hz vs. 1700 Hz, 1500 Hz vs. 1600 Hz, 1500 Hz vs. 1400 Hz, and 1500 Hz vs. 1300 Hz. Thirty-one females were used for the two-choice playback experiments. Various numbers of females did not respond to different tasks (4 in 1500 Hz vs. 1700 Hz, 7 in 1500 Hz vs. 1600 Hz, 11 in 1500 Hz vs. 1400 Hz, and 3 in 1500 Hz vs. 1300 Hz). These no-response cases were excluded from the statistical analyses in this section. Out of the four two-choice tasks, females showed a significant preference for the low dominant frequency call in only the 1300 Hz vs. 1500 Hz task (one-tail binomial test, *P* = 0.006, *N* = 28) and not the other three two-choice tasks (*P* ≥ 0.076) (Fig. 5).

## Discussion

The results of this study showed that both lek attendance and call dominant frequency were important in influencing the mating success of male *Z. prasinatus*. Males that (1) had higher lek attendance and (2) produced advertisement calls with lower dominant frequencies had a higher mating success than males that had lower lek attendance and produced higher frequency calls. Our two-choice playback experiments further confirmed that females were more attracted to lower dominant-frequency calls. The results of the study also showed that (1) males with better body condition and larger body sizes had higher lek attendance, (2) males with larger body size produced lower frequency calls, and (3) males’ body size had a marginally non-significant positive association with mating success. Together, these results indicate that both endurance rivalry and female choice play important roles in sexual selection in this treefrog population, and these two mechanisms appear to reinforce one another, resulting in selection for males with larger body size. We discuss these results below.

### Both endurance rivalry and female preference are important to male mating success

That lek attendance predicted mating success (rhacophorid treefrog: [60]; hylid treefrogs: [18, 19, 51]) and that better-conditioned males had higher lek attendance (*Hyla gratiosa*: [52]) are consistent with the findings in other frogs. Because body size and body condition are often good indexes of energy reserves [61, 62], the results of our study suggest that attending the chorus is energetically costly and represents an energetic constraint for male *Z. prasinatus*. In the barking treefrog (*H. gratiosa*), for instance, (1) males that spent more nights attending choruses had better initial but poorer final body condition than males that spent fewer nights attending choruses, and (2) males that were fed crickets as they left the chorus returned sooner and for more nights than did unfed males, showing attending choruses to be energetically costly and constrained in males [52]. According to the endurance rivalry hypothesis, male lek attendance reflects male quality [19]. Better quality males have higher energy reserves which enable them to attend and display in leks for longer periods. Furthermore, better quality males have also been found to incur the energetic costs more slowly. In barking treefrogs, males with longer chorus tenures lost condition less rapidly [52]. In male natterjack toads (*Bufo calamita*), males with larger body size lost their weight less rapidly (lower % weight lost) during breeding seasons [63]. Additionally, larger male treefrogs *Hyla arborea* were found to have lower mass-specific oxygen consumption rates during calling [64]. The advantage of having higher initial energy reserves and incurring energetic costs more slowly allows larger males or those with better condition to attend and display in leks for longer, which leads to higher mating success.

According to the endurance rivalry hypothesis, male lek attendance reflects male quality. Because good quality males are able to attend leks more frequently, females have a higher chance of mating with good-quality males and passing on the good genes to their offspring even when they mate randomly [19]. There are ample other examples of a positive association between lek attendance and male quality [19, 52]. In the European treefrog (*Hyla arborea*) [19] and the Omei treefrog (*Z. omeimontis*) [60], for example, lek attendance was positively correlated with male inter-annual survival. Evidence for males with higher lek attendance producing offspring with better quality and/or higher fitness is, however, more limited. A recent study of the Italian treefrog (*Hyla intermedia*) did show that the offspring of males with higher lek attendance have higher growth rates and metamorphose at younger ages [65], lending support to the hypothesis that lek attendance reflects male genetic quality. More studies on whether male lek attendance predicts the quality and fitness of their offspring will allow us to have a better understanding of whether it is a common phenomenon for females to gain indirect (genetic) benefits from mating with males with high lek attendance.

Out of the 10 call properties examined in our study, dominant frequency not only highly correlated with body size but also predicted male mating success. Together with the result of the playback experiments that females were more attracted to lower (than average) frequency calls, these results suggest that the dominant frequency of male advertisement calls served as an honest signal of male size to females in *Z. prasinatus*. In anurans, because the frequency of advertisement calls depends on the vibration of their vocal cords, and the size and mass of their laryngeal apparatus correlate positively with their body size [37, 66], larger males tend to produce calls with lower frequencies [40, 67]. Preferably mating with males that produce lower-frequency calls would thus allow females to find larger mates [49, 68]. For instance, in *Physalaemus pustulosus* [48] and *Hyla chrysoscelis* [49, 69], male call frequency negatively correlated with their SVL; females were more attracted to calls of lower frequencies and males with larger SVL had higher mating success. Body size is a good indicator of male quality because larger males tend to grow faster and/or survive longer [45, 46]. Studies have also shown that females benefit from mating with larger males by producing heavier offspring [70, 71].

Although males with larger SVL tended to have higher lek attendance and to produce calls with lower dominant frequencies, there was no significant relationship between male lek attendance and dominant frequency. Males that had higher lek attendance, therefore, did not necessarily produce lower frequency calls. Lek attendance and dominant call frequency thus appear to be two somewhat independent performance traits that have an additive influence on male mating success in *Z. prasinatus*. Our study, however, found a positive relationship between lek attendance and call rate. A similar relationship has also been found in other frogs [18]. Both attending leks more often [36, 52] and calling at higher rates [43] are energetically demanding. The positive relationship, again, indicates a lack of negative tradeoff between attendance and call effort in the males and suggests that males of better quality attend the lek more often and call at higher rates. Despite its positive association with lek attendance, call rate did not predict mating success in the males. Call rate was found to be positively associated with not only lek attendance but also male mating success in other species (e.g., the Italian treefrog) [18]. For our study, we were only able to record the calls of 24 males and only 7 out of these males were observed to have mated. The small sample size could have limited our ability to detect important relationships between call properties (other than dominant frequency) and male mating success. Furthermore, repeated measures of the call properties of the same males over the entire breeding season would allow more reliable evaluations of the relationships between different call properties and lek attendance and mating success.

In the playback experiments in our study, females consistently showed a preference for lower-frequency calls, although the difference reached significance only for the 1500 Hz vs. 1300 Hz choice task. The results of two-choice playback experiments in anurans differ between studies. Some species show preference for calls with lower frequency which suggests call frequency to be under directional selection and important for mate-quality assessment (*Hyla ebraccata*: [39]; *Physalaemus pustulosus*: [48]). Other species show preference for calls with a frequency close to the population mean which suggests call frequency to be under stabilizing selection and important for species recognition (green treefrog *Hyla cinerea*: [72]; the Italian treefrog *Hyla intermedia*: [73]). The results of both the field observations and the two-choice playback experiments in our study suggest the call dominant frequency of the males in our study population to be subject to directional selection from female choice.

In anurans, in addition to attracting breeding females, advertisement calls can also function to warn off conspecific rival males [36, 37]. The two functions in anurans are often achieved with the same calls (monophasic) [74, 75], but are effected by two different types of notes (diphasic) in some species [76]. In *Geocrinia victoriana*, for instance, males produce diphasic advertisement calls; the first phase of the call (“introductory notes”) is directed at other males in a territorial context, while the second phase (short “repeated notes”) functions primarily to attract conspecific breeding females [76]. In this study, we examined the importance of the call properties of type A notes to attracting females in male *Z. prasinatus* and showed males that produced lower frequency calls have higher mating success. We have never observed fights between males. It, however, remains possible that males could communicate with each other through their calls. Whether, and if so how, male calls play an important role in male-male interactions *Z. prasinatus* requires further investigation.

### Body size only non-significantly predicted mating success despite its strong association with both lek attendance and call frequency

Our results showed the body size and body condition of male *Z. prasinatus* to be strongly associated with lek attendance and the dominant frequency of the advertisement calls. Males with larger body size or better body condition were able to spend more nights displaying in the leks. Additionally, males with larger body size were also able to produce lower frequency calls. Because both lek attendance and the dominant frequency predicted mating success, we would have expected both endurance rivalry and female preference to result in selection for males with larger body size and better body condition. However, body size only non-significantly predicted mating success, and the positive relationship between body condition and mating success was virtually undetectable. These results are, however, consistent with the finding that lek attendance predicts mating success in many other lek-chorusing frogs and body size does not (reviewed in [19]). These results are also consistent with the results of a meta-analysis [77] which showed (1) the relationship between male call frequency and male body size to be the strongest, (2) the relationship between female preference and male call frequency to be of intermediate strength and (3) the relationship between female preference (i.e., mating success) and male body size to be the weakest. The authors explained that a weak preference-size relationship is to be expected because the relationship arises as an indirect consequence of the other two relationships [77].

Because lek attendance and call dominant frequency are also affected by factors other than body size and condition, choosing males based on these traits does not always allow females to find males with the largest body size or best body condition. For instance, in barking treefrogs, although body condition had a strong influence on the number of nights males attended choruses, mortality was suggested to account for the short tenures of some males because an estimated 20% of males died while calling in the chorus [52]. Therefore, endurance rivalry would select for males with an optimal combination of body condition and longevity. The importance of male mortality to lek attendance in our *Z. prasinatus* population is not clear, although we did observe many incidences of the frogs being eaten by the red-banded snake *Lycodon rufozonatum*, the bamboo pit viper *Trimeresurus stejnegeri* and the pointed-scaled pit viper *Protobothrops mucrosquamatus* in our study site.

Although call dominant frequency is generally a good indicator of body size, it can also be influenced by other factors. In the Brazilian hylid frog (*Hypsiboas atlanticus*), for example, the acoustic parameters of males’ advertisement calls were affected not only by their body size but also by the microhabitat; the dominant frequency was lower when they called with their body partially submerged in water than when they called perched on vegetation [78]. Male southern brown tree frogs (*Litoria ewingii*) at sites with more traffic noise called at a higher pitch [79]. Overall, the sensitivities of call properties (including dominant frequency) to environmental factors could reduce the accuracy of females selecting larger males through the properties of their calls. At our study site, males call in mixed species assemblages with high levels of background noise and in diverse microhabitats such as grass, shrubs, tree roots or tree tops. These factors could affect the ability of females to select for larger males based on the properties of their calls. Call properties most consistently and reliably associated with male quality should therefore play more important roles in female choice. This hypothesis, however, remains to be tested.

## Conclusions

Chorus attendance and call dominant frequency have additive influences on the mating success of male emerald treefrog. Body size affects both chorus attendance and call frequency, while body condition affects only chorus attendance but not call frequency. Because chorus attendance affects mating success, selection for males that attend the chorus often may indirectly result in selection for large males in good condition. Female preference for lower-frequency calls could further contribute to the selection for large males, but the importance of the contribution depends on the extent to which females are able discern differences in call parameters in natural choruses.

## Methods

### Study animals and study site

The emerald treefrog (*Z. prasinatus*) is a large rhacophorid frog, around 50–80 mm in SVL, and is endemic to northern Taiwan [54–56]. Although they can breed all year round, most reproductive activities (reproductive peaks) occur in the spring (March to April) and the fall (September to November) [54]. This study was conducted at the Hua-Lin experimental forest of Chinese Culture University, New Taipei City, Taiwan (24°54′ N, 121°34′E, 200-600 m a.s.l., 92 ha). The forest consists of secondary hardwood with patches of grassland. There are 16 rectangular (approximately 600 L each) and 1 round (4264 L) artificial concrete pools and eight abandoned plastic tanks (85 L each) in the forest which *Z. prasinatus* frequently uses for oviposition. The operational sex ratio is skewed toward males, and polyandrous mating has been observed in the *Z. prasinatus* in the forest [54]. During our study period, we did not observe peripheral males to kick the primary male down from the female in polyandrous mating, although such events are possible.

### Field survey

We surveyed our study site three to four nights per week (from 18:00 to 4:00 next morning) from September to December in 2016 (37 nights in total) and three nights per week from September to November in 2017 (25 nights in total). We visually searched for the frogs around the concrete pools and plastic tanks with flashlights and captured the frogs by hand. Because we sampled three to four (instead of seven) nights per week, there should be more frogs in the study site than were caught. For the frogs that we caught, we used a vernier caliper (Mitutoyo 500–700-10 dial caliper) to measure their snout-vent length (SVL) (to the nearest 0.01 mm), a portable electronic balance (Jadever JKD-500) to measure their body mass (to the nearest 0.1 g) and toe-clipping to mark them for individual identification [80, 81]. We recorded the ID of the males that were seen to be calling. For the oviposition events observed, we captured all of the frogs in the mating group, marked the unmarked individuals, measured their SVL and body mass, recorded the ID of the primary and peripheral males and then released them.

#### Males’ mating success, lek attendance and morphological traits

A male was classified as a primary male if it was observed to be the only male in monandrous mating or the first male to grasp the female in polyandrous mating. A male was classified as a peripheral male if it was only observed to be involved in mating events by jumping on amplectant pairs/groups in polyandrous mating. A male that was never observed to be involved in any mating events was an unmated male. A primary male acquires 100% paternity in monandrous mating and probably the majority of the paternity share (> 70% in a closely related species *Z. omeimontis*) in polyandrous mating [58]. We therefore considered the mating success to be the highest for the primary males, followed by the peripheral males and then the unmated males. Male lek attendance was calculated as the number of nights the male was observed calling at a lek divided by the number of survey nights (2016: 37 nights, 2017: 25 nights). If an individual was caught and measured more than once during the study period, the mean SVL and the largest body mass were used for all statistical analyses. Body condition was calculated as the residuals of a male’s largest body mass regressed on its (mean) SVL, which has been shown to be a good indicator of lipid storage in amphibians [62].

#### Male calls

During our 2017 field survey we recorded males’ advertisement calls using a directional microphone (Sennheiser ME67) and a digital recorder (Marantz PMD670) when it was not raining. When we located a calling male, we placed a directional microphone (mounted on a tripod) approximately 1 m away from the male and used a digital recorder to record its calls (44.1 kHz sampling rate, 16-bit resolution). Afterwards, we captured the male, measured its SVL and body mass and recorded its ID. Immediately after we captured the male, we also recorded the air temperature at the male’s calling site to the nearest 0.1 °C using a digital temperature monitor (Wisewind 5330).

**Fig. 6:**
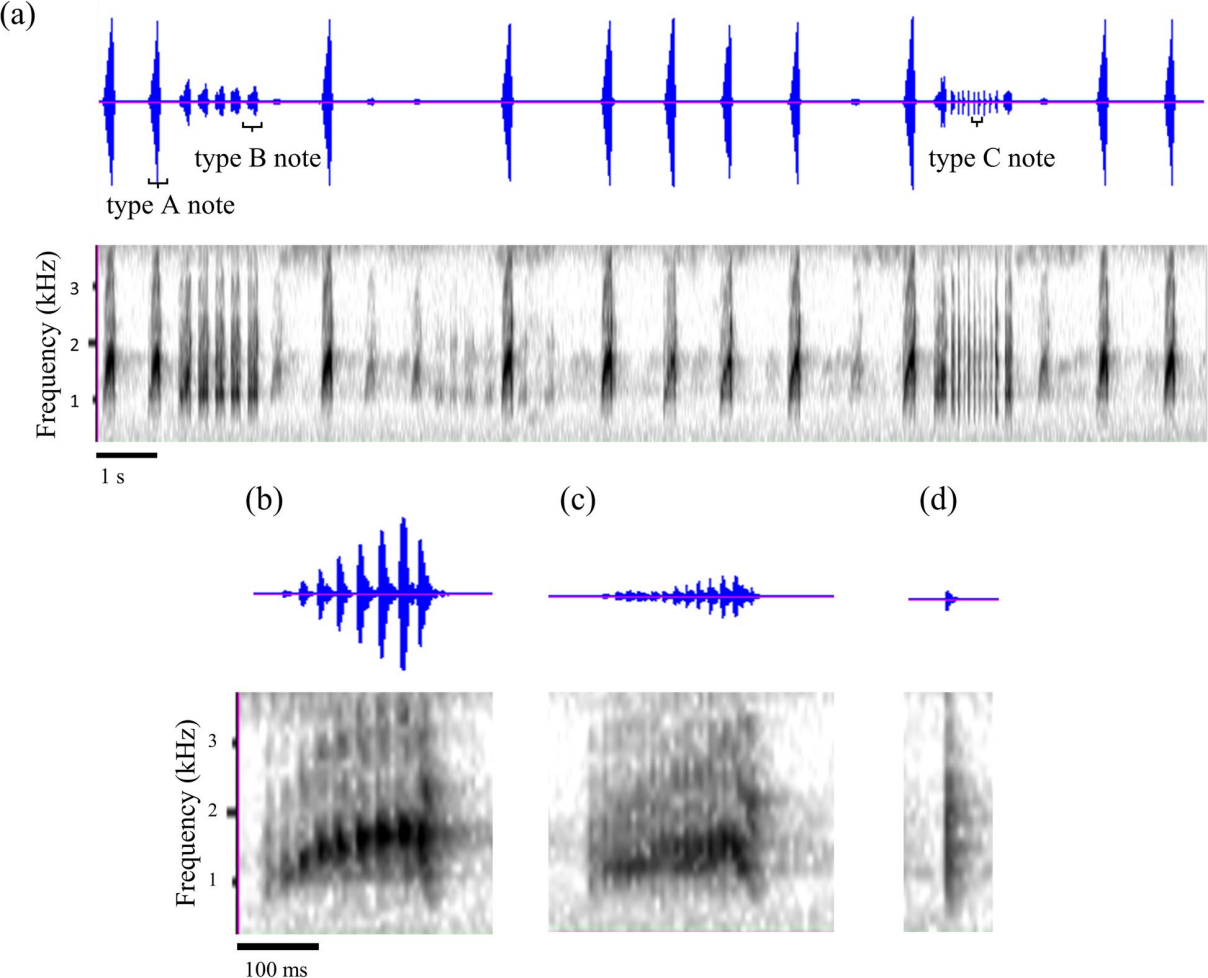
Waveforms (top) and spectrograms (bottom) of male emerald treefrogs’ (*Z. prasinatus*) advertisement call. **a** A section of the advertisement call that contains 3 types of notes: **b** type A, **c** type B and **d** type C

The male advertisement calls are comprised of various numbers of repeated notes. A note is a short series of pulses (1–12 pulses) produced in rapid succession [82]. Males of *Z. prasinatus* produce three types of notes (types A, B and C; Fig. 6a-d) [82]. A Type A note consists of six to nine pulses with note duration between 0.134 and 0.304 s and a dominant frequency between 1205 and 1722 Hz. Type A notes show clear frequency modulations (FM): the dominant frequency is lower in the first pulse, increases to the highest in the max pulse and then rapidly decreases in the final pulse (Fig. 6b). A type B note consists of three to twelve pulses with note duration between 0.065 and 0.248 s and a dominant frequency between 983 and 1554 Hz (Fig. 6c). A type C note consists of only one pulse and thus has very short note duration of 0.003 to 0.020 s, and has a dominant frequency between 947 and 1722 Hz (Fig. 6d). The males start their advertisement calls with a series of type A notes. Some males produce a series of type B (1 to 8 notes) and/or type C notes (3 to 10 notes) in-between type A notes.

We successfully recorded advertisement calls containing more than 12 type A notes from 24 males. Each of the 24 males was recorded once; previous studies also recorded all or a large proportion of males once when examining the relationships between call characteristics and male mating success [41, 50]. The calls of six of these 24 males comprised all three types of notes, the calls of 11 of the males comprised two types of notes (types A and B) and the calls of seven of the males comprised only type A notes. Males that produced one (type A), two (types A and B) or all three types (types A, B, and C) of notes did not differ in their mating success (i.e., the likelihood of being the primary, peripheral or unmated males) (*χ*^2^_4_ = 6.85, *N* = 24, *P* = 0.144). All males produced type A but not type B or C notes, indicating type A notes to be essential to their call displays. We therefore analyzed only the properties of type A notes. We quantified ten call properties (Additional file 1: Fig. S1) [83–85] for each of the type A notes using Raven Pro v1.3 (Bioacoustic Research Program 2014). For temporal properties, we quantified (1) note duration: the duration between the onset and the offset of a note, (2) note rate: the reciprocal of the note period (note period: time from the onset of a note to the onset of the following note), (3) note interval: the time between the offset of a note and the onset of the following note, (4) rise time: the time between the onset of a note and the maximum waveform in a note, (5) fall time: the time between the maximum waveform in a note and the offset, (6) pulse number: number of pulses in a note and (7) pulse rate: the number of pulses divided by note duration. For the spectral properties, we quantified (8) dominant frequency: the frequency that carries the most energy in a note. In addition, we quantified (9) FM-rise: dominant frequency in max pulse minus dominant frequency in the first pulse and (10) FM-fall: dominant frequency in final pulse minus dominant frequency in max pulse because we found the dominant frequency in type A notes to have periodic rising (early) and falling (late) frequency modulation (FM). For the 10 call properties for a male, we quantified the 10 properties from 12 to 20 type A notes of its advertisement calls and calculated the medians for each of the call properties.

### Female preference experiments

The results of the 2017 field survey showed males’ mating success to be significantly associated with the dominant frequency of the advertisement calls but not the other 9 properties: males that produced calls with lower-frequency type A notes had higher mating success. We subsequently conducted two-choice playback experiments using synthesized calls to test whether females indeed show preference for calls with lower dominant frequencies.

#### Experimental design and procedures

The two-choice playback experiments were modified from Rosso et al. [73]. For each of the choice tasks, a female was presented with a pair of digitally synthesized call stimuli which differed in dominant frequency but not temporal properties (i.e., note duration and note rate) (see ‘*Synthesis of the call stimuli*’ below). The means (± standard deviations) of the dominant frequencies of the type A notes from the 24 males’ calls recorded in 2017 was 1500 (± 100 kHz). Based on these results, we designed four two-choice tasks examining females’ preference between calls with the mean dominant frequency (1500 Hz) and calls with a higher or lower dominant frequency (± 1 or 2 SD): 1500 Hz vs. 1700 Hz, 1500 Hz vs. 1600 Hz, 1500 Hz vs. 1400 Hz, and 1500 Hz vs. 1300 Hz. Each of the test females was exposed to these four two-choice tasks in random order.

We built an indoor semi-anechoic chamber (length × width × height: 3 m × 3 m × 2 m) using a stainless steel frame and covered the top and the four sides with five pieces of sound-absorbing foam. Inside the chamber, we used a black plastic frame (height: 30 cm) and a black screen to designate a circular arena (diameter: 2.7 m) (Additional file 1: Fig. S2). We placed two speakers (Fujitsu Multimedia Speaker System PS-150) at opposite corners of the chamber immediately outside the arena (Additional file 1: Fig. S2). The two speakers were randomly assigned to play the two different call stimuli of a task in randomly assigned order. The average sound pressure level (SPL) 1 m away from the 24 calling males we recorded was 83 dB. Before each trial, we used a sound level meter (TMS TES-1350A) to measure the SPL 1 m away from the speakers and then adjusted it to 83 dB. All the task trials were conducted in the dark. We monitored and recorded all task trials with an infrared night vision camera (Mi Home Security Camera SXJ01ZM) mounted on the underside of the cover of the chamber.

The experiments were conducted between February and May, 2019. The test females used for the experiments were caught from the study site on the night of the experiments. After being captured, a female was placed in a plastic acclimatization container (l × w × h: 37 × 23.5 × 15 cm; with a water basin and a tree branch inside) for 1 h before being used in the experiments. We cut an opening (10 × 15 cm) on the lid for ventilation and covered the opening with a mesh screen. To make sure that females were sexually responsive, we captured only females in amplexus that had not yet spawned. After the 1-h acclimatization, we removed the female from the container, placed it inside an acoustically transparent cylindrical plastic cage (37 cm tall, 16 cm in diameter) with a top opening. We then placed the cage at the center of the arena and placed a plastic cover over the top opening (Additional file 1: Fig. S2). The plastic cover was attached to a string which we could pull to remove the cover remotely. We played back the predesignated pair of call stimuli for 3 min before remotely removing the cover of the cage to start a task trial and allow the female to move freely. The playbacks continued throughout the entire trial. A test female was deemed to show a preference if, within 10 min of the start of a task trial, it moved within 10 cm of one of the two speakers (inside the preference area in Additional file 1: Fig. S2). If a test female did not show preference in 10 min, the task trial was terminated. After a task trial was completed or terminated, the female was placed back in the acclimatization container for 30 min before the next task trial. After all the four task trials were completed, we toe-clipped the females and released them to their capture sites.

#### Synthesis of the call stimuli

We first randomly selected five males from the 24 males that were recorded in 2017. From the calls of each of these males, one type A note was then randomly chosen to serve as the template to generate synthetic calls (a total of five templates). To synthesize the call stimuli for a test female, we randomly picked one note template and used the digital audio editor Audacity (Portable 2.3.2) to adjust the dominant frequency to generate notes with the five dominant frequencies for the experiments (1700, 1600, 1500, 1400 and 1300 Hz). We used the digital audio editor GoldWave (Portable 6.45) to adjust the duration of these notes to 0.187 s (the mean of the note duration of the calls of the 24 males recorded in 2017). We then used the digital audio editor Audacity (Portable 2.3.2) to replicate these notes to generate call stimuli consisting of 13 min of repetitive type A notes with a note rate of 0.65 notes/s (the mean note rate of the calls of the 24 males recorded in 2017); each call stimulus consisted of type A notes of one specific dominant frequency. This note rate allowed the notes of the pair of call stimuli to not overlap when played back from the two speakers during the playback experiments. The call stimuli of five dominant frequencies that a test female was exposed to were synthesized from the same note template.

### Statistical analysis

We used simple ordinal logistic regression models to test, separately, the importance of males’ body size (i.e., SVL), body condition and lek attendance to their mating success (i.e., the likelihood of being the primary, peripheral or unmated males). We used Spearman correlation coefficients (*r*_*s*_) to examine the significance of the correlations between males’ lek attendance and their morphological traits (SVL and body condition) because the distribution of lek attendance was very skewed.

We used simple linear regression models to investigate the relationship between call properties and ambient temperature because temperature affects acoustic properties of advertisement calls in anurans [37]. In our data, the duration, rise time and pulse rate of type A notes were significantly associated with ambient temperature (Additional file 1: Table S1). We therefore adjusted these three call properties to an ambient temperature of 22 °C (= mean temperature of survey nights) using the regression coefficients (= original value - *b* × (ambient temperature of the male’s calling site - 22 °C)) [18]. For these temperature-dependent call properties, the adjusted values (duration_adj_, rise time_adj_ and pulse rate_adj_) were used in subsequent analyses. The mean ± SE and the range of the 10 call properties are provided in Table S2 (Additional file 1). We used simple ordinal logistic regression models to evaluate, individually, the importance of each of the males’ 10 call properties to their mating success. We used Pearson’s correlation coefficients (*r*) to examine the significance of the correlations between males’ call properties and their morphological traits (SVL and body condition). We used Spearman correlation coefficients (*r*_*s*_) to examine the significance of the correlations between males’ call properties and their lek attendance because the distribution of lek attendance was very skewed.

For the two-choice playback experiments, we used one-tailed binomial tests to examine whether the test females showed a preference for the lower dominant frequency of type A notes for each of the four two-choice tasks.

We used JMP pro 14 (SAS Institute Inc., Cary, NC, USA) for all statistical analyses.

## Supplementary Information

**Additional file 1: Table S1.** The effect of ambient temperature on call properties. Simple linear regression models (a-j) evaluating the effect of ambient temperature on each of the 10 call properties. *N* = 24 for all models. *P* values ≤0.05 are marked in bold. **Table S2.** Basic statistics of the 10 call properties. The mean ± SE and the range of the 10 call properties. *N* = 24. **Fig. S1.** Illustrations of the acoustic properties of the advertisement call of male *Z. prasinatus.* (a) A section of the advertisement call that contains 3 types of notes: A, B and C. (b) The temporal properties of a type A note. (c) The power spectrum of a type A note showing the dominant frequency of the note (i.e., the frequency corresponding to the largest energy peak). (d) Spectrograms showing frequency modulation (FM) in a type A note: the dominant frequency was lower in the first pulse and increased to the highest in the max pulse and rapidly decreased in the final pulse. **Fig. S2.** The setup of the semi-anechoic chamber for the two-choice playback experiments.

**Additional file 2.** An excel sheet file containing the dataset supporting the conclusions of this article.

## Data Availability

The dataset supporting the conclusions of this article is in an additional file (Additional file 2).

## Abbreviations

SVL: Snout-vent length
FM: Frequency modulation
