# Supplementary material for: Endurance rivalry and female choice jointly influence male mating success in the emerald treefrog (Zhangixalus prasinatus), a lek-chorusing anuran
Source: BMC Zool. 2022 Apr 1;7:17. doi: 10.1186/s40850-022-00117-w (PMC10127438; doi:10.1186/s40850-022-00117-w)
Supplement: Supplementary file 1 — Additional file 1: Table S1. The effect of ambient temperature on call properties. Simple linear regression models (a-j) evaluating the effect of ambient temperature on each of the 10 call properties. N = 24 for all models. P values ≤0.05 are marked in bold. Table S2. Basic statistics of the 10 call properties. The mean ± SE and the range of the 10 call properties. N = 24. Fig. S1. Illustrations of the acoustic properties of the advertisement call of male Z. prasinatus. (a) A section of the advertisement call that contains 3 types of notes: A, B and C. (b) The temporal properties of a type A note. (c) The power spectrum of a type A note showing the dominant frequency of the note (i.e., the frequency corresponding to the largest energy peak). (d) Spectrograms showing frequency modulation (FM) in a type A note: the dominant frequency was lower in the first pulse and increased to the highest in the max pulse and rapidly decreased in the final pulse. Fig. S2. The setup of the semi-anechoic chamber for the two-choice playback experiments. [file 40850_2022_117_MOESM1_ESM.docx]

**Supplementary Tables and Figures**

Title: Endurance rivalry and female choice jointly influence male mating success in the emerald treefrog (*Zhangixalus prasinatus*), a lek-chorusing anuran

Yuan-Cheng Cheng, Yi-Huey Chen, Chunwen Chang, Ming‐Feng Chuang, Yuying Hsu

**Table S1 The effect of ambient temperature on call properties.**

Simple linear regression models (a-j) evaluating the effect of ambient temperature on each of the 10 call properties. *N* = 24 for all models. *P* values ≤ 0.05 are marked in bold.

|  | **Call property** | ***b* ± SE** | ***F*** | ***P*** |
| --- | --- | --- | --- | --- |
| (a) | Duration | -0.009 ± 0.00 | 28.14 | **<0.001** |
| (b) | Rate | 0.011 ± 0.02 | 0.23 | 0.640 |
| (c) | Interval | -0.041 ± 0.05 | 0.71 | 0.408 |
| (d) | Rise time | -0.008 ± 0.00 | 31.31 | **<0.001** |
| (e) | Fall time | -0.000 ± 0.00 | 0.04 | 0.839 |
| (f) | Pulse number | 0.026 ± 0.06 | 0.20 | 0.657 |
| (g) | Pulse rate | 1.751 ± 0.16 | 124.94 | **<0.001** |
| (h) | Dominant frequency | 0.129 ± 7.13 | 0.00 | 0.986 |
| (j) | FM-rise | -3.459 ± 6.27 | 0.30 | 0.586 |
| (j) | FM-fall | -14.902 ± 8.55 | 3.04 | 0.095 |

**Table S2 Basic statistics of the 10 call properties.**

The mean ± SE and the range of the 10 call properties. *N* = 24.

| **Call property** | **Mean ± SE** | **Range** |
| --- | --- | --- |
| Duration_adj_ (s) | 0.19 ± 0.01 | 0.14-0.25 |
| Rate (1/s) | 0.65 ± 0.07 | 0.23-1.70 |
| Interval (s) | 1.69 ± 0.15 | 0.42-3.53 |
| Rise time_adj_ (s) | 0.14 ± 0.00 | 0.10-0.18 |
| Fall time (s) | 0.05 ± 0.00 | 0.03-0.07 |
| Pulse number | 7.08 ± 0.18 | 6.00-9.00 |
| Pulse rate_adj_ (number/s) | 38.93 ± 0.48 | 34.59-43.70 |
| Dominant frequency (Hz) | 1532.46 ± 21.98 | 1205.90-1722.70 |
| FM-rise (Hz) | 506.04 ± 19.43 | 258.65-689.00 |
| FM-fall (Hz) | -213.54 ± 28.10 | -430.70-0.00 |


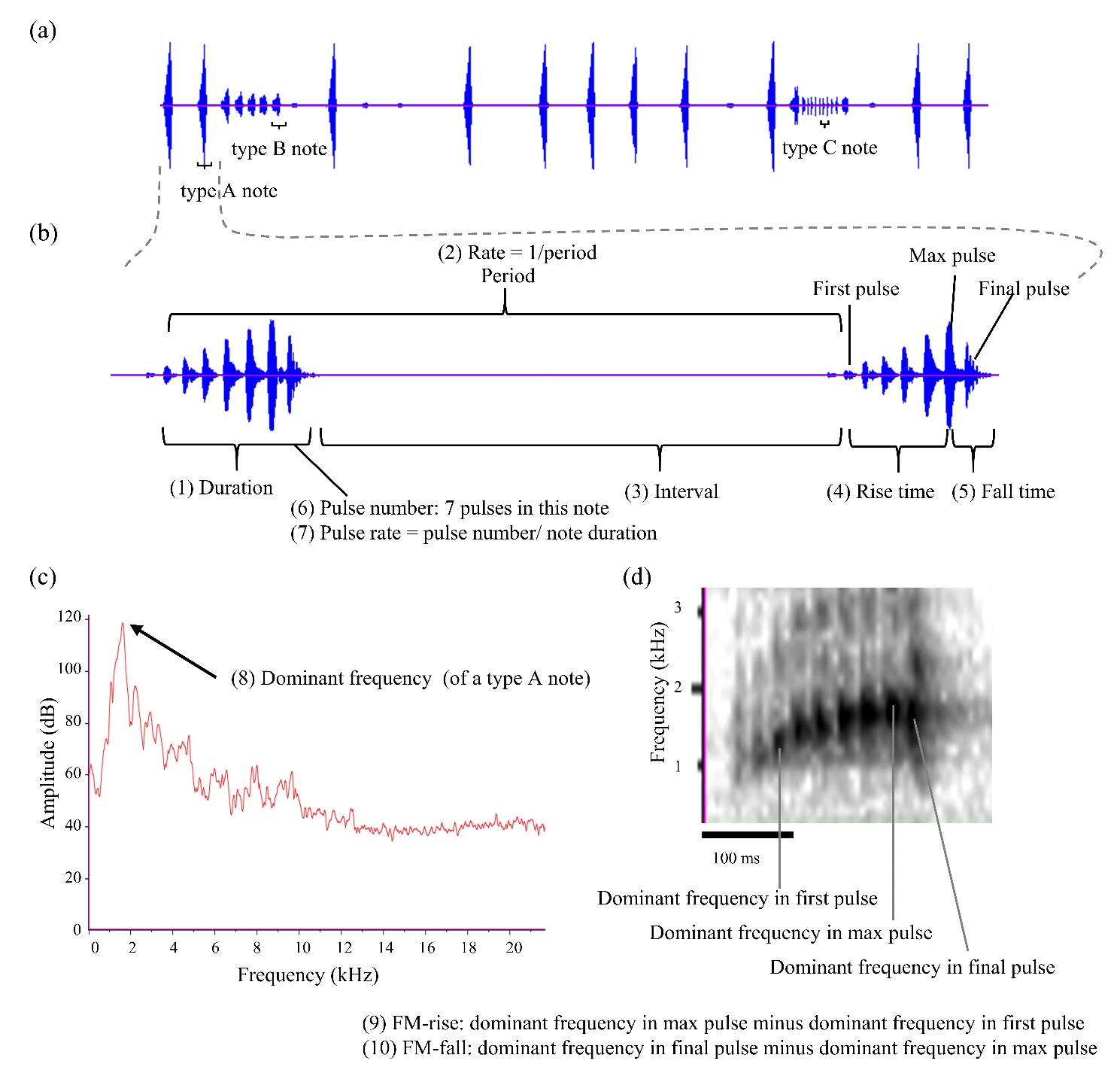


**Fig. S1 Illustrations of the acoustic properties of the advertisement call of male *Z. prasinatus.***

(a) A section of the advertisement call that contains 3 types of notes: A, B and C. (b) The temporal properties of a type A note. (c) The power spectrum of a type A note showing the dominant frequency of the note (i.e., the frequency corresponding to the largest energy peak). (d) Spectrograms showing frequency modulation (FM) in a type A note: the dominant frequency was lower in the first pulse and increased to the highest in the max pulse, and rapidly decreased in the final pulse.


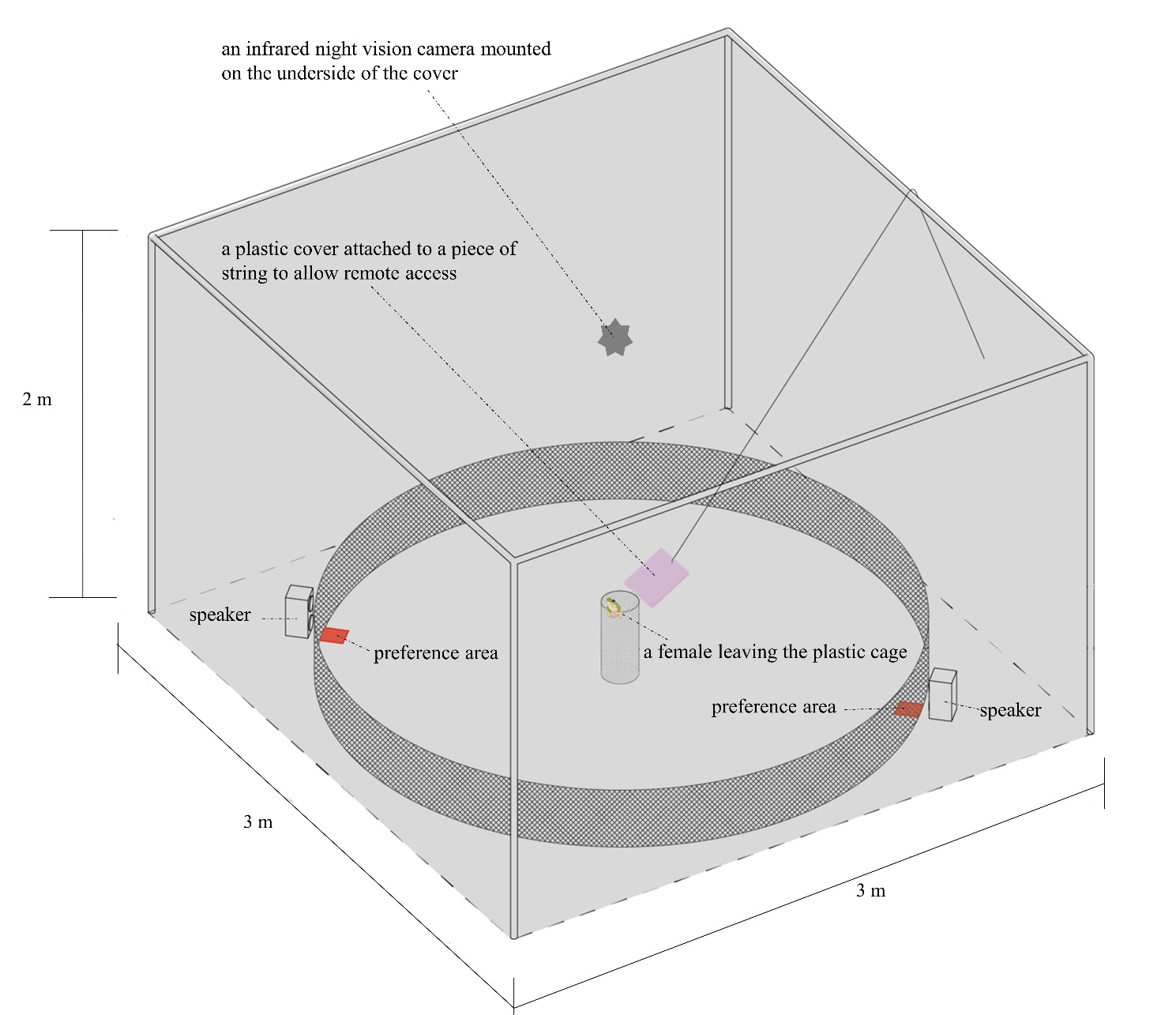


**Fig. S2 The setup of the semi-anechoic chamber for the two-choice playback experiments.**
